# Does health facility service environment matter for the receipt of essential newborn care? Linking health facility and household survey data in Malawi

**DOI:** 10.7189/jogh.07.020508

**Published:** 2017-12

**Authors:** Liliana Carvajal–Aguirre, Vrinda Mehra, Agbessi Amouzou, Shane M Khan, Lara Vaz, Tanya Guenther, Maggie Kalino, Nabila Zaka

**Affiliations:** 1 Data and Analytics, Data Research and Policy, UNICEF, New York, New York, USA; 2 Department of International Health, Johns Hopkins School of Public Health, Baltimore, Maryland, USA; 3 Saving Newborn Lives, Save the Children, Washington, DC, USA; 4 National Statistical Office, Malawi; 5 Program Division, UNICEF, New York, New York, USA

## Abstract

**Background:**

Health facility service environment is an important factor for newborns survival and well–being in general and in particular in high mortality settings such as Malawi where despite high coverage of essential interventions, neonatal mortality remains high. The aim of this study is to assess whether the quality of the health service environment at birth is associated with quality of care received by the newborn.

**Methods:**

We used data from the Malawi Millennium Development Goals Endline household survey conducted as part of MICS survey program and Service Provision Assessment Survey carried out in 2014. The analysis is based on 6218 facility births that occurred during the past 2 years. Descriptive statistics, bivariate and multivariate random effect models are used to assess the association of health facility service readiness score for normal deliveries and newborn care with newborns receiving appropriate newborn care, defined for this analysis as receiving 5 out of 6 recommended interventions during the first 2 days after birth.

**Results:**

Newborns in districts with top facility service readiness score have 1.5 higher odds of receiving appropriate newborn care (adjusted odds ratio (aOR) = 1.52, 95% confidence interval CI = 1.19–1.95, *P* = 0.001), as compared to newborns in districts with a lower facility score after adjusting for potential confounders. Newborns in the Northern region were two times more likely to receive 5 newborn care interventions as compared to newborns in the Southern region (aOR = 2.06, 95% CI = 1.50–2.83, *P* < 0.001). Living in urban or rural areas did not have an impact on receiving appropriate newborn care.

**Conclusions:**

There is need to increase the level of service readiness across all facilities, so that all newborns irrespective of the health facility, district or region of delivery are able to receive all recommended essential interventions. Investments in health systems in Malawi should concentrate on increasing training and availability of health staff in facilities that offer normal delivery and newborn care services at all levels in the country.

Recent evidence estimates that care around the time of birth including having a skilled attendant at birth, emergency obstetric care, immediate care for newborns, and newborn resuscitation could prevent 1.5 million maternal and newborn deaths and stillbirths by 2025 [1]. The days and weeks around childbirth and immediate postnatal period – are the most vulnerable for both mothers and newborns. Most maternal and infant deaths occur during this time [2]. Care during the time of labour, child birth and early, postnatal care (PNC), presents a unique opportunity to set both mothers and babies on a good start. Postnatal care also provides the delivery platform for care of the newborn, including the promotion of preventive practices and detection of any complications. Care of the normal newborn includes early initiation of (exclusive) breastfeeding, prevention of hypothermia, clean postnatal care practices and appropriate cord care [3]. Close observation for 24 hours and at least three additional postnatal contacts is recommended for all mothers and newborns to establish good caregiving practices and detect any life–threatening conditions [4]. However, for improved effectiveness, newborn care interventions in the postnatal period should be delivered as a package. Every Newborn Action Plan launched in 2014 to end preventable newborn deaths envisages each country to ensure 90% of all births receive quality care improve PNC coverage at least by 20% by 2020 and 90% by 2030. PNC is also a key indicator for EWEC monitoring framework which will facilitate monitoring of SDG targets by 2030 [3].

Addressing newborns’ health is a priority in Malawi as in many countries in sub–Saharan Africa. In 2015, newborns in Malawi accounted for 34% of all under–5 deaths, an increase from 2000 when newborns accounted for 20% of under–five deaths [5]. This increase in proportion of newborn deaths in overall under–five deaths speaks about the effect of immunization and reduction of diarrhoea and pneumonia related mortality. Malawi is one the few counties in sub–Saharan Africa which has reached the MDG goal 4 by reducing under–five deaths by 63% between 1990 and 2015. During the same period, the country also reduced its maternal mortality ratio by over one–third (34%) and witnessed a substantial increase in the rate of institutional deliveries; from 55% in 2000 to 91% in 2016 [6]. However, between 1990 and 2015, neonatal deaths have declined by only 36% (6). Additionally, recent data shows wide regional variations with regards to perinatal mortality rate. In 2016, the Central region had a perinatal mortality rate of 42 per 1000 pregnancies compared to 29 per 1000 in the Southern region [7]. The slower decline in newborn mortality relative to under–5 mortality in Malawi calls for a redoubling of efforts, including attention to premature babies and care for small and sick babies [8].

In Malawi, health care services are provided by three agencies; Government through the Ministry of Health (MoH) provides about 60%; the Christian Health Association of Malawi (CHAM) is responsible for about 39% plus a small contribution from the private–for profit health sector [9]. Attention to newborn health intensified after 2005 as the Government of Malawi integrated newborn survival and implemented the Essential Health Package and developed a multi–year national initiative (2005–2015), the ‘Road Map’ for Accelerating Reduction of Maternal and Newborn Mortality and Morbidity in Malawi [10]. Malawi Newborn Action Plan was developed and launched in 2015 and the country recently committed to WHO–UNICEF’s network for Improving Quality of Care for Maternal, Newborn and Child Health. Ministry of Health engaged NSO to conduct partner resource mapping exercise and results showed variations in terms of support on MNCH interventions including newborns. There was more concentration mostly on maternal issue than new born issues leading to verticalization in the implementation on newborn care either by partners or districts. As identified by health authorities in the country, challenges in Malawi remain both acute and complex with projections on human resources. To ensure adequate staffing at health facilities, in 2012 the Government implemented an “Emergency Human Resource” program for re–engagement and redeployment of staff [11]. This has not been implemented fully and the health sector strategic plan 2 (2017–2022) is carrying on this work. Still, at current output levels, it will take many years to come anywhere near the numbers of health staff needed to provide minimum standards of service delivery [12].

The quality and availability of health services that are within reach to mothers and newborns, the service environment, plays a major role in the provision of good care. The relationship between health services and population outcomes is an important area of public health research that requires bringing together data on health outcomes and the relevant health service environment [13]. However, as newborn health is relatively new on the global agenda, data on the service environment for this vulnerable group is still scarce [14]. Malawi presents a great opportunity to explore the convergence of complementary data on health facilities and population–based data on this topic as it is one of the few countries with census facility data and household survey data within a range of close years readily and publicly available for analysis.

An important additional consideration in many low–income settings is the distance to health facilities, particularly in rural areas as roads may not be optimal and vehicles for transport are rarely available.

Distance to delivery care and the level of care provided are important determinants of facility delivery [15] and thus of the well–being of mothers and babies. Recent studies in sub–Saharan Africa show a significant variation in receiving postnatal care. Across communities in Nigeria and Uganda [16] studies have found that distance to health facilities as well as socio economic factors are important determinants for accessing services [17,18]. Recent geospatial analysis have also identified that targeted interventions at the district level are essential to strengthen maternal health programmes [19,20]. This study investigates if living in a district with health facilities that are ready to provide a high level of normal delivery and newborn care is associated with receiving a package of essential newborn care interventions during the first two days after birth.

## METHODS

### Data

Two main sources of data have been used for this analysis: the Malawi MDG Endline Survey 2014 – MES conducted as part of the UNICEF supported MICS survey program [21] which is a population–based household survey representative at the national and district level, and the Malawi Service Provision Assessment 2013–2014 – MSPA 2014 [22], which is based on a census of health facilities in the country. To determine population densities across districts, we used census data from the Malawi 2008 census as 2013–14 projections were not available at the time of the analysis [23].

### Population based data

The Malawi MDG Endline Survey (MES) was carried out in 2013–14 by Malawi National Statistical Office as part of the global Multiple Indicator Cluster Survey (MICS) programme. Technical support was provided by the United Nations Children’s Fund (UNICEF). The sample for the MES 2014 was designed to provide estimates for a large number of indicators at the national level; for urban and rural areas; the three regions (Northern Region, Central Region and Southern Region); and the 27 districts of Malawi excluding the island of Likoma due to logistical challenges. The sample was stratified by district with the aim of obtaining representative estimates at each district level. Within each district, the sample was further stratified by urban–rural, before a two stage cluster sampling was implemented. Within each stratum, a specified number of census enumeration areas were selected systematically with probability proportional to size [21]. All the information obtained from respondents remains strictly confidential and anonymous. Although GPS coordinates of each sample cluster was collected, this information was not collected of respondents’ household.

In the MES 2014, a total of 24 230 women aged 15–49 years were interviewed between November 2013 and April 2014. Of the interviewed women, 31% had a live birth in the past two years, for a total of 7490 reported live births. Of these, 89% were born in health facilities (6661 live births). In the survey, women were asked questions about interventions related to maternal and newborn care that mothers and their newborns received immediately after birth and in the following few weeks. These questions include a number of critical interventions such as thermal care, feeding practices like early initiation of breastfeeding, weighing of the baby and more that are recommended to occur during the postnatal period to ensure the well–being of the baby [2]. Of the 6661 facility–based births in the last two years reported in the household survey, 6218 had complete data on all variables of newborn care and were included in the analysis.

### Health facility data

The Malawi Service Provision Assessment MSPA 2014 was implemented by the Malawi Ministry of Health. ICF International provided technical assistance through the MEASURE DHS program, which is funded by USAID and is designed to assist countries in collecting data to monitor and evaluate population, health, and nutrition programmes [22]. The MSPA 2014 is considered a census of facilities in the country as it covers all of Malawi’s health facilities including public and semi–public facilities of all levels, CHAM as well as major private facilities [22]. The survey assesses whether components considered essential for quality service delivery are present and functioning [22]. Data also includes precise location using GPS of all facilities in the country.

Of the 977 health facilities in Malawi, 528 (54%) were recorded as providing normal delivery and newborn care services and were included in the study. For this analysis, data from the MSPA 2014 facility and providers data sets were used. These modules collected information on basic emergency and neonatal services in key domains including: staff and training, equipment, and key medicines and commodities relevant during delivery and to provide care for the newborn. Variables about health facility services were ascertained through observation and health facility staff interviews, in the facility and providers data set of the MSPA 2014 [22]. No missing data was observed for the variables included from the MSPA 2014 facility and provider data sets in this analysis.

### Definition of outcome and exposure

#### Outcome: Appropriate newborn care

In 2013, WHO released the Postnatal Care for Mother and Newborn guidelines which provided a list of recommendations for the care of the mother and newborn in the postnatal period [2]. The specific recommendations for the newborn included assessment of the baby, exclusive breastfeeding, cord care and thermal care interventions. We recognize that the scope of newborn care in the postnatal period is broad and encompasses a range of interventions. But, for the purposes of this analysis, appropriate newborn care is defined as co–coverage of essential interventions received by the newborn in the period immediately after birth and up to 2 days after birth for which data was available in the Malawi MES 2014 survey. Thus, a newborn was considered to have received appropriate newborn care if he/she received 5 out of 6 of the following interventions: 1) being weighed after birth, 2) being put to the breast during the first hour after birth, 3) not having received pre–lacteal feeds, 4) being wiped/dried after birth, 5) being bathed not before 6 hours after birth, 6) having received a postnatal check within 2 days following birth. The interventions for immediate care for newborns selected in this analysis were also consistent with the recommendations in the Every Newborn Action Plan (ENAP), which at its onset provided evidence of the effectiveness of these interventions for improving newborn survival [1]. The “appropriate newborn care” score was calculated using equal weights for each of the six components (Table S1a in **Online Supplementary Document**). The present analysis focused only on normal newborns and did not include premature, sick babies requiring additional interventions.

#### Exposure: Facility level readiness score

**Figure 1:**
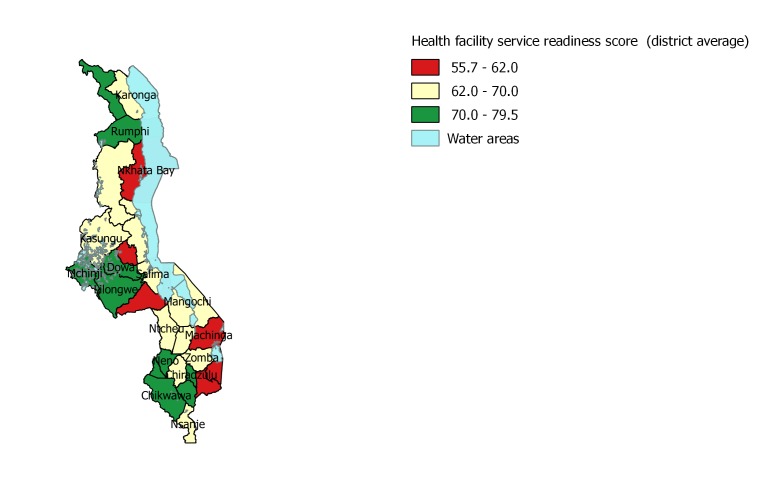
District level ‘normal delivery and newborn care’ health facility service readiness score.

The quality of delivery and newborn care services offered in health facilities are characterized by calculating the service readiness score for “normal delivery and newborn care” based on the Service Availability and Readiness Assessment (SARA).”*– Reference Manual* [24]. The score includes three domains: 1) staff and training: having guidelines for integrated management of pregnancy and childbirth (IMPAC) and having staff trained in IMPAC. IMPAC was selected as the Malawi service provision assessment reports on IMPAC as the guidelines for facilities offering normal delivery service [22] 2) equipment and commodities (observed and functioning), and 3) medication and supplies availability. A total of 20 tracer indicators were included in the construction of the score (Table S1b in **Online Supplementary Document**), covering the 19 SARA tracer indicators plus having an infant scale given its relevance to the outcome under investigation. Facility specific scores ranged from 19 to 100. These scores were then aggregated at the district level using weighted average and the final scores were not stratified by the type of facility. To account for facility utilization, district level scores were weighed by the number of outpatient clients in each facility. District level service readiness scores ranged from 56 to 80 with a mean of 67.1. For ease of interpretation, these were then categorized into terciles based on their mean value: bottom (55.7–62), middle (62–70) and top (70–79.5) (**Figure 1**).

### Method of analysis

To investigate the association between appropriate newborn care for newborns and district average facility service readiness score, the two data sources were linked using the administrative boundary linking method. This approach consists of linking the two data sets at a level at which both are representative [25]. Thus, following this method, facility data were aggregated at the district level and then merged with the individual level household survey data set for subsequent analysis. Recent analysis linked health facility and household survey using this same method for the analysis of availability of improved water and sanitation in the childbirth environment in 58 countries [26]. The study undertook an ecological type of analysis where facility births in a particular district were assigned their respective district average health facility score. Thus, each of the 6218 facility births from the individual data set included in the analysis was assigned a district average health facility score value according to their district location.

Bivariate regression analyses of potential confounders related to household, mother, delivery and infant (Table S1a in **Online Supplementary Document**) were analysed for association with the primary outcome of appropriate newborn care. Variables found to be significant in the bivariate analysis were selected for inclusion in the final model. A random effect multiple logistic model was used to assess the association between the variables of interest. This implies that levels of ‘appropriate newborn care’ in neighbouring districts are unrelated after adjusting for other variables in the model. This modelling technique was used given the structure of the data and to account for the effects of clustering. All variables kept in the model were checked for multicollinearity by assessing variance inflation factor. Analysis was conducted in Stata 14.0 [27] and maps were produced in QGIS desktop 2.14.0. [28].

### Ethical approvals

All data are publicly available and therefore no ethics approval was required for this analysis. Ethical approval for data collection was the responsibility of data collectors.

## RESULTS

### Descriptive analysis

Table S2 in **Online Supplementary Document** presents the distribution of health facilities with service–readiness score (70 or above) in the top category in each domain. This descriptive analysis reveals that 52% of the facilities in urban areas have a service readiness score of 70 or higher, in contrast to rural areas where only 32% of the facilities recorded high scores. The results are further disaggregated by regions and districts. The domain with the lowest performance is staff and training. Across districts, there is a wide range in the proportion of facilities with a high score on this domain (range: 0–55%; mean 21.7%). For the equipment and supplies domain, the range is 25.2 to 77.6% (mean 46.7%) of facilities with scores in the top category. For the medicines and commodities domain, the range is 20.0 to 81.8% (mean 54.5%) with score 70 or higher. Across all districts, 35.3% of facilities (range 16.8 to 66.8%) have a service readiness score of 70% or higher. **Figure 1** presents the mean district health facility score.

At the individual level, of the 6218 facility births, 37% were in districts in which the average facility service readiness score was above 70%. Of all newborns included in the analysis, 88% were located in rural areas and 12% in urban areas, 14% were born to mothers younger than 20 years old, for 82% their mothers had either no education (11%) or only primary education (71%). Of the 6218 births, 86% were delivered in public health facilities (**Table 1**).

**Table1:** Distribution of study population characteristics – live births in facilities in the past 2 years and crude associations with outcome (N = 6218)

Indicators	Total n (%)	Prevalence >5 newborn care, n (%)	Unadjusted OR (95% CI)	*P*
**Health facility readiness score (district average):**				<0.001
Bottom (55.7–62)	2117 (34)	1714 .0)	1	
Middle (62–70)	1813 (29)	1570 (86.6)	1.52 (1.21–1.91)	<0.001
Top (70–79.5)	2288 (37)	1994 (87.2)	1.60 (1.25–2.03)	<0.001
**Place of residence:**				
Urban	745 (12)	663 (88.9)	1	
Rural	5473 (88)	4615 (84.3)	0.67 (0.50–0.90)	<0.001
Region:				
Southern	3018 (48)	2457(81)	1	
Central	2467 (40)	2165 (87.7)	1.63 (1.32–2.02)	<0.001
Northern	733 (12)	656 (89.5)	1.95 (1.32–2.90)	<0.001
**Mother’s age at birth (years):**				
<20	850 (14)	707 3.3)	1	
20–34	4617 (74)	3841 (85.1)	1.14 (0.85–.152)	0.374
35–49	852 (14)	729 (85.6)	1.18 (0.84–1.68)	0.329
**Mother’s education:**				
None	664 (11)	542 1.6)	1	
Primary	4387 (71)	3722 (84.8)	1.27 (0.97–1.65)	0.080
Secondary or higher	1168 (19)	1014 (86.9)	1.49 (1.05–2.12)	0.025
**Household wealth index:**				
Poorest	1464 (24)	1203 .2)	1	
Second	1389 (22)	1176 (84.7)	1.19 (0.91–1.58)	0.200
Middle	1290 (21)	1093 (84.7)	1.20 (0.88–1.64)	0.240
Fourth	1059 (17)	911 (86.1)	1.34 (0.96–1.86)	0.081
Richest	1017 (16)	894 (87.9)	1.58 (1.12–2.22)	0.008
**Type of health facility:**				
Public health facility	5348 (86)	4563 (85.3)	1	
Private health facility	194 (3)	157 (81.1)	0.74 (0.43–1.26)	0.271
CHAM Mission	676 (11)	558 (82.5)	0.81 (0.60–1.09)	0.171
**Type of delivery:**				
Vaginal delivery	5032 (81)	5032 (85.3)	1	
C-Section	245 (4)	244 (76.3)	0.55 (0.39-0.79)	0.001
**Parity (number of children):**				
1 child	1466 (24)	1216 2.9)	1	
2-3 children	2293 (37)	1966 (85.7)	1.24 (0.96–1.60)	0.101
4–5 children	1483 (24)	1292 (87.1)	1.39 (1.08–1.79)	0.011
6+ children	975 (16)	804 (82.4)	0.96 (0.71–1.30)	0.809
**Baby size:**				
Not very small	6008 (97)	5107 (85.0)	1	
Very small	210 (3)	171 (81.2)	0.76 (0.49–1.18)	0.224
**Density of facilities with score above 70%:**				
Below mean	3820 (61)	3215 .2)	1	
Above mean	2398 (39)	2062 (86.0)	1.15 (0.95–1.40)	0.148

CHAM – Christian Health Association of Malawi, CI – confidence interval, OR – odds ratio

### Bivariate analysis

**Figure 2:**
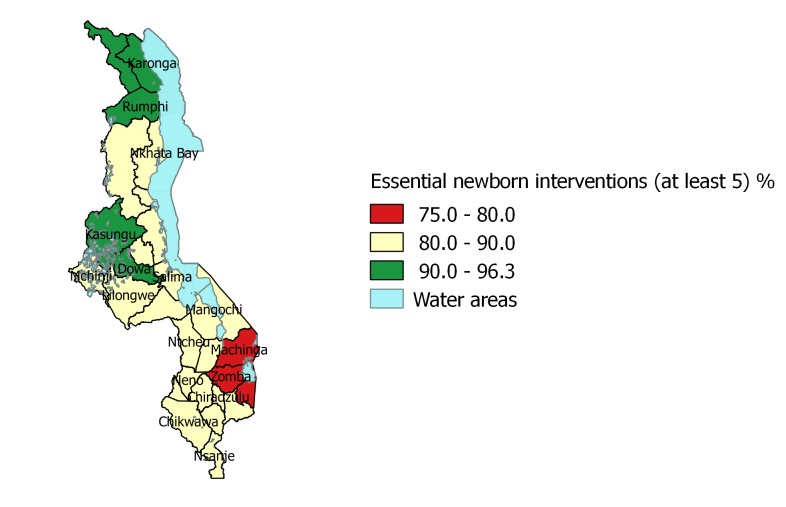
Coverage of appropriate essential newborn care.

Analysis of essential newborn care interventions across regions found that the Southern region presents lower coverage of newborns receiving all 6 newborn care interventions (37.1% CI = 34.4–39.9) (Table S3 in **Online Supplementary** **Document**). The interventions with significant differences in coverage across regions are: early initiation of breastfeeding, newborns being bathed 6 hours after birth or later and newborns receiving essential newborn care visit within 2 days. In terms of the combined ‘appropriate newborn care’ variable, in the Northern region nearly 90% (89.5% CI = 85.5–92.6) of the newborns received at least 5 newborn care interventions followed by Central (87.7%, CI = 85.8–89.4, *P* < 0.001) and Southern regions (81.4%, CI = 79.4–83.3, *P* < 0.001). Coverage of all 6 of the essential newborn interventions is considerably lower across all regions. While half of all newborns (49.5%) received all 6 interventions in the Central region, coverage was recorded at 41.1% in the Northern region and 37.1% in Southern region. These unadjusted distributions take account of the complex survey design but do not consider clustering, therefore should be interpreted with caution. **Figure 2** presents coverage of the appropriate newborn care interventions measured at the district level.

The crude analysis using simple logistic regression, presented in **Table 1**, shows a positive association between appropriate newborn care and service readiness facility score of 70 and above (OR = 1.60, 95% CI = 1.25–2.03, *P*<0.001). Other variables found to be associated with appropriate newborn care are: residence, region, mother’s education (secondary or higher), household wealth (fifth quintile), delivery by c–section and parity (having 4–5 children). For instance, newborns in the Northern and Central region of the country (as compared to newborns in the Southern part), newborns whose mothers have secondary or higher education (as compared to mothers with no education), newborns in households in the highest wealth quintile (as compared to the lowest wealth quintile) had higher likelihood of receiving appropriate newborn care in the postnatal period. On the other hand, newborns in rural areas and newborns who were born by c–section had lower likelihood of receiving at least 5 newborn care interventions immediately after birth. There was no evidence of significant association between appropriate newborn care and mother’s age at birth, type of health facility, baby size at birth or density of facilities with high score.

### Multivariate analysis

The final model testing the association between receipt of appropriate newborn care and health facility service readiness score was adjusted for region, residence, household wealth, mother’s education, type of delivery and baby’s size at birth and residence. The results of the fully adjusted random effect logistic model are presented in **Table 2**.

**Table2:** Association between appropriate newborn care and district level health facility score for “normal delivery and newborn care” – random effect logistic model (N = 6218)

Variable	Categories	Adjusted OR (95%CI)	*P*
Health Facility Readiness Score	Bottom	1	
	Medium	1.29 (0.98–1.69)	0.067
	Top	1.52 (1.19–1.95)	0.001
Region	Southern	1	
	Central	1.53 (1.20–1.95)	0.001
	Northern	2.06 (1.50–2.83)	<0.001
C–Section	No	1	
	Yes	0.55 (0.42–0.73)	<0.001
Baby size at birth	Other	1	
	Very small	0.60 (0.43–0.84)	0.003
Household Wealth Index	Poorest	1	
	Second	1.20 (0.98–1.47)	0.070
	Middle	1.22 (0.99–1.50)	0.057
	Fourth	1.26 (1.01–1.59)	0.044
	Richest	1.37 (1.02–1.84)	0.036
Mother’s education	None	1	
	Primary	1.21 (0.87–1.50)	0.091
	Secondary	1.40 (1.05–1.86)	0.023
Place of residence	Urban	1	
	Rural	0.84 (0.63–1.13)	0.254
Random effect variance (σ)		0.195	0.003
ICC (ρ)		0.011	

OR – odds ratio, CI – confidence interval, ICC – intra–cluster correlation coefficient

The analysis reveals that newborns in districts with a facility score in the top category have 52% increased odds of receiving appropriate newborn care (OR = 1.52, 95% CI 1.19–1.95, *P* = 0.001), compared to newborns in districts with a facility score in the bottom category. Co–variates with a statistically significant positive association with newborns receiving at least 5 newborn care interventions are: region, household wealth, mother’s education. Newborns residing in the Northern region are two times more likely to receive 5 essential newborn care interventions as compared to newborns in the Southern region (OR = 2.06, 95%CI 1.50–2.83, *P* < 0.001); the odds for newborns living in the Central region are increased by 1.5 as compared to newborns in the Southern region (OR:1.53, 95% CI 1.20–1.95, *P* = 0.001), having a mother with secondary or higher education increases the odds of better essential newborn care by 1.4 (OR = 1.40, 95% CI 1.05–1.86, *P* = 0.023) and by 1.37 if living in a household in the highest wealth quintile (OR = 1.37, 95% CI = 1.02–1.84, *P* = 0.036). On the other hand, newborns delivered by c–section (OR = 0.55, 95% CI 0.42–0.73, *P* < 0.001) and very small babies (OR = 0.60, 95% CI 0.43–0.84, *P* = 0.003) have lower odds of receiving the type of essential newborn care analyzed in this study. This may be due to the fact that the postnatal care protocol for c–section and low birth weight babies is different [29]. Unexpectedly, even though residing in rural areas showed a significant effect in the crude analysis, once the model was fully adjusted this effect was lost (OR = 0.84, 95% confidence interval 0.63–1.13, *P* = 0.254).

The cluster measures calculated in the model are the random effect variance (sigma = 0.195), which measures the in–between cluster variation and the intra–cluster correlation coefficient ICC (ρ = 0.011). These results give an indication of clustering within districts given that the values are not zero. The random effect variance is significant (*P* < 0.003). In other words, the result of the test of the null hypothesis of no within–district clustering provides strong evidence of within–district clustering in the model. Thus, it can be assumed that districts contributed to explain the variance in receiving appropriate newborn care.

## DISCUSSION

This analysis investigated whether health facility service readiness score for normal delivery and newborn care at the district level is associated with receiving appropriate newborn care in the postnatal period in Malawi. The results indicate that newborns in districts with average facility service readiness score in the top category (score or 70% or higher) have 52% increased odds to receive appropriate newborn care than those in districts with lower facility score. The role of location is highlighted in the results as newborn in the Northern region have 2 times increased odds to receive appropriate newborn care as compared to newborns in the Southern region. As recent research has identified, addressing geographic and quality barriers is crucial to enhance service utilization and to lower maternal and perinatal mortality [30].

As reported in MSPA 2014 report, coverage of essential newborn care interventions is particularly high across health facilities in Malawi [22]. However, geographic location plays a role in the observed level of coverage disparity and health service environment. The level of health facility readiness to provide normal delivery and newborn care varies across the country (score range 56 to 80%) and only 35% of facilities across the country have a readiness score higher than 70%. A particular concern is that staff and training, which is a key domain of the health facility service readiness score is the lowest across the country. For instance, very few facilities in the Southern region have a score higher than 70% on the staff and training domain.

There are important limitations in this analysis that should be considered when interpreting the results. The measure of appropriate newborn care is based on a sub–set of recommended interventions for normal newborns for which data was available in the MES 2014 survey. Further, data on newborn care interventions is based on mother’s recall of care provided to the newborn soon after birth. As with other measures based on mother’s recall, this could have led to differential recall bias and may not entirely reflect the level of quality of care in facilities [31,32]. This is particularly the case for interventions that occur during the postpartum period as it is an extremely intense moment for mothers. Some mothers may not be aware of what is going on with their newborn given factors related to the dynamics of labour and delivery such as tiredness and soreness after labour, medical complications, or just the excitement of receiving their new child into the world.

Another limitation is the unavailability of GPS data at the cluster level which did not allow assessment of location within districts and distance to facilities. As the 2014 MES survey did not collect GPS data, the smallest level of aggregation possible was the district level. Districts have a number of facilities that provide different level of services. An average of the facility score at the district level may be an oversimplification of the reality. In addition, since districts are the lowest common level of geographic aggregation between the two data sets used in the analysis, further investigation of the effect of clustering at a lowest level of data collection (enumeration areas/clusters) was not possible and therefore would be a great choice for further research. To link the population and health facility data, a number of important assumptions were made. For instance, mean district health facility service readiness scores have been assigned to districts where the respondent resided at time of interview. However, this is a considerable limitation as with the available data it cannot be determined if a woman delivered in her own district or in a health facility outside of her district.

Given that this is a cross–sectional study, a cause–effect relationship cannot be established. It was also not possible to adjust for other potential confounding factors in the final model not available in the MES 2014. For instance, distance to health facility, motivation or awareness of mothers and health staff of essential newborn care procedures, availability of roads and transportation to access health facility, family support, the quality of the actual services received, women’s autonomy, etc. A major confounder which could not be assessed in this study is the presence of a strong component of community–based maternal and newborn care in Malawi. For example: Ministry of Health revised the 2 week training on Community Based maternal and new born care to a 6–day training to increase coverage and improve access of these services. This process has so far covered almost 50% of the districts in the country. Supervision and mentorship tools have also been developed to support and strengthen implementation at district level [33].

Despite these limitations, the study provides evidence that the geographic proximity to facilities that provide optimal delivery and newborn care services can have an impact on the number of essential services received by the newborn. The main strengths of this analysis are the linking of health facility data with household survey data that allowed for joint analysis of health service environment and coverage indicators. Analysing these two sources of data also allowed for the inclusion of confounders at the individual, household and facility level. An important aspect of the analysis was that it looked into the quality of health facilities by analysing 3 main aspects important for normal deliveries and newborn care: staff and training, medicines and commodities as well as equipment and supplies. Previous quantitative studies have linked facility data and household data from DHS [15,34]. This methodology has a strong bearing on quality of care measurements which can use measures of essential newborn care interventions in household surveys as predictors of facility readiness.

## CONCLUSION

The analysis reveals that in Malawi, newborns in districts with higher health facility service readiness score have increased odds of receiving a more complete set of essential newborn care interventions compared to those residing in districts with a lower facility score. These variations in readiness among geographical areas require a focused programming in order to address newborn care problems and achieve the targets that were set in the Every Newborn Action Plan. Therefore, it is imperative to increase the level of service readiness across all facilities, so that newborns regardless of the place and type of facility delivery receive all recommended essential interventions.

Staff availability and training emerged as an issue across all the districts in the country that can negatively affect the services received by newborns. Our study results suggest that given limited resources, priority should be given to high volume facilities in the poorly performing districts in the southern and northern regions. The essential newborn care interventions assessed in this study can for the most part be implemented with basic equipment available in most facilities and thus improvement strategies will need to address facility staff knowledge and motivation and other barriers including inadequate staffing levels. Strengthening of supervision, provision of simple job aids/checklists around essential newborn care, and ensuring adequate staffing for delivery and newborn care should be explored. In February 2017, the Malawi Ministry of Health joined eight other countries in launching a network for improving quality of care for maternal, newborn and child health and established a Quality of Care Management Directorate focussed on improving quality of care. Results of this study can help guide priority setting around what are the critical factors for the provision of quality services particularly in a context of a high neonatal mortality setting as Malawi [33]. Similarly, tracking of progress from mapping exercise and human resource development plan should be given attention as it will be critical for improvements in newborn care service delivery.

Over the last decade, the Government of Malawi has undertaken major initiatives to strengthen maternal and newborn care and improve staffing levels at health facilities. Getting performance reports and results from implementation of the current tools and instruments following the revised new born care guidelines will be a necessary as the Ministry looks forward for future domestic and international investments in health systems in Malawi. Additionally, it is critical to continue to analyse available data to generate evidence that will lead to the development of evidence based and focused programming for newborn care to the required standards in all parts of the country.
